# How Can We Make Decision for Patients With Chronic Hepatitis B According to Hepatitis B Virus (HBV) DNA Level?

**DOI:** 10.5812/hepatmon.15285

**Published:** 2014-02-05

**Authors:** Maryam Keshvari, Seyed Moayed Alavian, Heidar Sharafi

**Affiliations:** 1 Blood Transfusion Research Center, High Institute for Research and Education in Transfusion Medicine, Tehran, IR Iran; 2 Middle East Liver Disease (MELD) Center, Tehran, IR Iran; 3 Baqiyatallah Research Center for Gastroenterology and Liver Diseases, Baqiyatallah University of Medical Sciences, Tehran, IR Iran

**Keywords:** Hepatitis B, Liver Cirrhosis, Viral Load

## Abstract

**Background:**

HBeAg negative hepatitis B infection exerts both inactive carrier state and chronic active hepatitis, which are sometimes difficult to differentiate. Serial hepatitis B virus (HBV) DNA quantification, alanine transaminase (ALT) measurement, and liver histology assessment can help to differentiate these forms of hepatitis B infection.

**Objectives:**

We aimed to clarify the clinical and laboratory characteristics of HBeAg negative hepatitis B patients.

**Patients and Methods:**

Patients with hepatitis B, referred to Tehran Blood Transfusion Hepatitis Clinic from 2011 to 2013, were included and followed for one year. Laboratory assessments including liver function tests, HBV DNA quantification, and liver biopsy (for some cases) were performed.

**Results:**

Two hundred forty-three HBeAg negative hepatitis B patients were stratified into three groups based on to their HBV DNA level including group 1 (G1) with HBV DNA level < 2000 IU/mL, group 2 (G2) with HBV DNA level 2000-20000 IU/mL, and group 3 (G3) with HBV DNA level > 20000 IU/mL. The G2 had more similarity to G1 than G3 regarding their clinical characteristics.

**Conclusions:**

It is concluded that most HBeAg negative hepatitis B patients with serum HBV DNA level of 2000-20000 IU/mL, persistent normal ALT concentration, and no or mild liver damage on biopsy can be clinically managed as HBV inactive carriers.

## 1. Background

Hepatitis B virus (HBV) infection is a significant health problem. Approximately 350 million persons worldwide are chronically infected with HBV. Patients with chronic HBV infection are at increasing risk of end stage liver disease such as cirrhosis and hepatocellular carcinoma (HCC). According to different studies, 15% to 40% of HBV infected patients develop serious complications of hepatitis B (1, 2). The prevalence of HBV infection in Iran was estimated to be 2.14% (3). All HBV isolates from Iran were genotype D (4, 5). The rate of precore and basal core promoter mutations and progressive liver disease in patients with HBV genotype D infection was found to be more prevalent than HBV infected patients with non-D genotypes (6, 7). Hepatitis B infection consists of few clinical phases including immune tolerance phase, immune clearance phase, and inactive carrier state. Inactive carrier state is characterized by normal alanine transaminase (ALT) concentration, absence of HBeAg and presence of anti-HBeAb, low or undetectable HBV DNA in serum, and minimal or no histologic changes on liver biopsy. Some patients continue to have moderate levels of HBV replication and active liver disease, but remain to have negative results for HBeAg which most of them have the HBV variants that cannot produce HBeAg due to mutations in the basal core promoter or precore regions (8). According to the last guideline for management of hepatitis B, absence of HBeAg, presence of anti-HBeAb, Serum HBV DNA level < 2000 IU/mL, persistent normal ALT/aspartate transaminase (AST) level, and absence of significant hepatitis on liver biopsy are classified as inactive carrier state. Whereas, HBeAg positivity or serum HBV DNA level > 20000 IU/mL, persistent or intermittent elevation in ALT/AST level, and liver biopsy showing chronic hepatitis with moderate to severe necro-inflammation are classified as chronic active hepatitis B (9). This classification is important since antiviral therapy must be considered just for the patients with chronic active hepatitis presentation. However, differentiation between HBeAg negative chronic active hepatitis B and inactive carrier state is often challenging. We have observed some patients with HBV infection in the clinic with normal ALT levels, and HBV DNA levels between 2000 IU/mL and 20000 IU/mL and no or minimal fibrosis on their liver biopsy. Classification of this group of HBV infected patients into chronic active hepatitis or inactive carrier state is a great question.

## 2. Objectives

The aim of this study was to precise assessment of HBeAg negative hepatitis B infected patients according to their clinical and laboratory features.

## 3. Patients and Methods

In the current study, a total of 243 treatment naive HBV infected patients referred to Tehran Blood Transfusion Hepatitis Clinic (Tehran, Iran) from 2011 to 2013 were included and followed for one year. HBeAg positive chronic hepatitis B patients and cases with human immunodeficiency and hepatitis C and D antibodies were excluded. Laboratory assessments including liver function tests and HBV DNA quantification (HBV DNA level) were performed for the study population on at least two consecutive patients’ samples in a one-year interval. Upper normal limit of ALT was considered 34 IU/L for non-overweight women (BMI of less than 25), and 40 IU/L for non-overweight men (10). HBV DNA level using COBAS TaqMan HBV tests (Roche Diagnostics) and liver biopsy (The results were reported according to the modified Knodell scoring system, as the Ishak score.) was assessed. On liver histology, liver fibrosis score (stage) ≤ 2, and liver necro-inflammation score (grade) ≤ 4 were considered as cutoff values to show mild liver damage (11). The liver histology assessment was performed for patients with clinical and laboratory evidence of progressive liver disease, while it was performed optionally for those without such evidence. All study participants provided informed consent, and the study design was approved by appropriate ethics review board. Statistical analysis was performed using SPSS version 20. Categorical variables and continuous variables were analyzed by Fisher exact test and t-test, respectively. P values less than 0.05 were considered to be statistically significant. 

## 4. Results

In the current study, 243 HBeAg negative hepatitis B patients were divided into three groups regarding their HBV DNA level including group1 (G1) with HBV DNA level < 2000 IU/mL, group2 (G2) with HBV DNA level between 2000 IU/mL to 20000 IU/mL, and group3 (G3) with HBV DNA level > 20000 IU/mL. We observed that G2 had more similarity to G1 than G3 (Table 1). There was no statistically significant difference regarding sex, necro-inflammatory score on liver biopsy (grade), and total bilirubin concentration between these groups (P > 0.05), but there were significant differences in liver fibrosis (stage), AST, ALT and direct bilirubin mean concentrations between G2 and G3 (P < 0.05) (Table 1). 

**Table 1.** Comparison of Demographic and Clinical Characteristics of the Study Population Stratified by HBV DNA

	HBV DNA < 2000 IU/mL (G1) (n = 104)	HBV DNA 2000-20000 IU/mL (G2) (n = 53)	HBV DNA > 20000 IU/mL (G3) (n = 86)	P value G1 vs. G2	P value G2 vs. G3
**Age, Mean ± SD, y**	47.4 ± 12.7	41.8 ± 12.8	43.5 ± 13.9	0.01 ^a^	0.46^ a^
**Sex, No. (%)**					
Female	19 (18.3)	16 (30.2)	22 (25.6)	0.11^b^	0.56^b^
Male	85 (81.7)	37 (69.8)	64 (74.4)		
**Liver Fibrosis, No. (%)**					
Mild	16 (94.1)	29 (87.9)	41 (56.2)	0.65^b^	< 0.01 ^b^
Moderate to severe	1 (5.9)	4 (12.1)	32 (43.8)		
**Liver Necro-Inflammation, No. (%)**					
Mild	12 (80.0)	14 (48.3)	30 (42.3)	0.06^b^	0.66^b^
Moderate to severe	3 (20.0)	15 (51.7)	41 (57.7)		
**ALT ** ^**c**^ **, Mean ± SD, IU/L**	40.3 ± 28.7	40.5 ± 27.0	68.5 ± 53.1	0.98^a^	< 0.01^a^
**AST ** ^**c**^ **, Mean ± SD, IU/L**	29.7 ± 17.1	32.1 ± 18.6	46.9 ± 26.5	0.42^a^	< 0.01^a^
**Direct bilirubin, Mean ± SD, mg/dL**	0.23 ± 0.14	0.31 ± 0.19	0.42 ± 0.39	< 0.01^a^	0.03^a^
**Total bilirubin, Mean ± SD, mg/dL**	0.92 ± 0.54	1.08 ± 0.58	1.24 ± 0.89	0.10^a^	0.26^a^

^a^ t-test.

^b^ Fisher exact test.

^c^ Abbreviations: ALT, alanine transaminase; AST, aspartate transaminase.

## 5. Discussion

A previous study showed that HBV DNA level more than 20000 IU/mL can be considered as a cutoff value to differentiate patients with HBeAg negative chronic active hepatitis B and those in an inactive carrier state (12). However, another study proposed that due to the fluctuation in HBV DNA level among HBeAg negative chronic hepatitis B patients, there is no absolute cutoff value reliable to differentiate HBeAg negative chronic active hepatitis B patients and hepatitis B inactive carriers (13). Another study confirmed that ALT ≤ 30 IU/L and HBV DNA load ≤ 20000 IU/mL had high sensitivity, specificity, positive predictive value (PPV), and negative predictive value (NPV) to differentiate HBV inactive carriers and HBeAg negative chronic active hepatitis B (14). In a cohort of Alaska natives, among HBeAg negative hepatitis B patients, 25% met the criteria for chronic active HBV infection. The patients had mild histologic liver changes, if HBV DNA level never exceed 20000 IU/mL during the follow up period (15). A study from Greece showed that histologically significant liver disease was rare in patients with HBV infection with persistent normal liver enzymes, and HBV DNA ≤ 20000 IU/mL, so these patients could be considered as true inactive HBV carriers (16). On the other hand, in different studies, HBV DNA level of 2000 IU/mL was considered to differentiate inactive carriers and HBeAg negative chronic hepatitis B (17, 18). However HBV DNA level can fluctuate to lower than 2000 IU/mL in patients with HBeAg negative chronic hepatitis B, and as a result HBV clinical state can be misclassified based on a single HBV DNA level, so appropriate follow-up by HBV DNA and ALT levels is recommended to differentiate inactive carriers and patients with HBeAg negative chronic hepatitis B (18). One of our study limitations was its proportionally small number of patients with HBV DNA level 2000 IU/mL to 20000 IU/mL. We propose a longitudinal study with larger sample size to seek the outcome of this group of patients.

In conclusion, it seems that patients with HBV infection should be followed regularly with ALT and HBV DNA levels assessment. The current study, demonstrated that most HBeAg negative hepatitis B patients with serum HBV DNA level between 2000 IU/mL to 20000 IU/mL, persistent normal ALT concentration, and no or mild liver damage on biopsy could be considered as HBV inactive carriers.
